# *In-Vitro* Archaeacidal Activity of Biocides against Human-Associated Archaea

**DOI:** 10.1371/journal.pone.0062738

**Published:** 2013-05-03

**Authors:** Saber Khelaifia, Jean Brunel Michel, Michel Drancourt

**Affiliations:** 1 Aix Marseille Université, URMITE, UMR63 CNRS 7278, IRD 198, Inserm 1095, Marseille, France; 2 Centre de Recherche en Cancérologie de Marseille (CRCM), CNRS, UMR7258; Institut Paoli Calmettes; Aix-Marseille Université, UM 105; Inserm, U1068, Marseille, France; University of Groningen, Netherlands

## Abstract

**Background:**

Several methanogenic archaea have been detected in the human intestinal microbiota. These intestinal archaea may contaminate medical devices such as colonoscopes. However, no biocide activity has been reported among these human-associated archaea.

**Methodology:**

The minimal archaeacidal concentration (MAC) of peracetic acid, chlorhexidine, squalamine and twelve parent synthetic derivatives reported in this study was determined against five human-associated methanogenic archaea including *Methanobrevibacter smithii*, *Methanobrevibacter oralis*, *Methanobrevibacter arboriphilicus, Methanosphaera stadtmanae*, *Methanomassiliicoccus luminyensis* and two environmental methanogens *Methanobacterium beijingense* and *Methanosaeta concilii* by using a serial dilution technique in Hungates tubes.

**Principal Findings:**

MAC of squalamine derivative S1 was 0.05 mg/L against *M. smithii* strains, *M. oralis*, *M. arboriphilicus, M.* *concilii* and *M. beijingense* whereas MAC of squalamine and derivatives S2–S12 varied from 0.5 to 5 mg/L. For *M. stadtmanae* and *M. luminyensis*, MAC of derivative S1 was 0.1 mg/L and varied from 1 to ≥10 mg/L for squalamine and its parent derivatives S2–S12. Under the same experimental conditions, chlorhexidine and peracetic acid lead to a MAC of 0.2 and 1.5 mg/L, respectively against all tested archaea.

**Conclusions/Significance:**

Squalamine derivative S1 exhibited a 10–200 higher archaeacidal activity than other tested squalamine derivatives, on the majority of human-associated archaea. As previously reported and due to their week corrosivity and their wide spectrum of antibacterial and antifungal properties, squalamine and more precisely derivative S1 appear as promising compounds to be further tested for the decontamination of medical devices contaminated by human-associated archaea.

## Introduction

An increasing number of methanogenic archaea are being found in the human microbiota [1]. Since Miller and collaborators reported the isolation of methanogenic archaea *Methanobrevibacter smithii* [2] from human feces, new strains were recently identified. Thus, *Methanosphaera stadtmanae* [3] and *Methanomassiliicoccus luminyensis* were isolated from human feces [4] and *Methanobrevibacter oralis* was identified from the human subgingival plaque [5]–[7]. Recently, we isolated *Methanobrevibacter arboriphilicus* [8] and *Methanobrevibacter millerae* [9] from human feces specimens (S. Khelaifia, M. Drancourt, unpublished data). Whereas *M. smithii* is an almost constant inhabitant of the human gut [10], *M. stadtmanae* was only found in about one-third of individuals [10] and *M. luminyensis* in an average of 4% individuals with an age-dependent prevalence [11]. It has been shown that purge used prior to colonoscopy, may not eliminate these particular methanogenic archaea: for instance, halophilic and methanogenic archaea *M. smithii, M. stadtmanae, M. arboriphilicus* and *Methanosaeta concilii* [12] were detected in colonic mucosal biopsies from patients who had received a purge [13]. Therefore, these human-associated archaea may contaminate any medical device soiled by feces such as colonoscopes. This contamination could be problematic because archaea significantly differ from bacteria so that the archaeacidal activity of biocides cannot be simply deduced from their bactericidal activity. Moreover, human-associated archaea have been found to be highly resistant to most commonly used antibiotics [14], [15]. In the perspective of broadening the spectrum of new active molecules, and more precisely against archaea organisms colonizing the human gut, squalamine and its derivatives appear to be among the few antimicrobial agents able to demonstrate an efficient anti-archaea activity [14], [15]. Squalamine is a natural aminosteol compound which is extracted from the spiny dogfish shark live [16]. Among various properties, it was found to be a potent antimicrobial compound active against both Gram-positive and Gram-negative bacteria and fungi [17]. We previously observed its in-vitro activity against four methanogenic archaea [15]. Here, we investigated the *in-vitro* archaeacidal activity of 15 biocides including original squalamine derivatives against seven archaea including five human-associated archaea.

## Materials and Methods

### Archaea

*M. smithii* ATCC 35061T DSMZ 861, *M. smithii* DSMZ 2374, *M. smithii* DSMZ 2375, *M. smithii* DSMZ 11975, *M. oralis* DSMZ 7256 T, *M. stadtmanae* ATCC 43021T DSMZ 3091, *Methanobacterium beijingense* [18] DSMZ 15999 and *M. concilii* DSMZ 2139, purchased from the German Collection of Microorganisms and Cell Cultures (DSMZ, Braunschweig, Germany). *M. arboriphilicus* strain tested in this study was recently isolated in our laboratory from human feces (S. Khelaifia, M. Drancourt, unpublished data). *M. smithii* strains, *M. arboriphilicus* and *M. beijingense* were grown on liquid media 119 (http://www.dsmz.de). The media 119 modified by addition of 1 g of Yeast extract and 2.5-bar of H_2_/CO_2_ (80/20) atmosphere was used to cultivate *M. oralis.* The media 322 (http://www.dsmz.de) was used to cultivate *M. stadtmanae* and the media 334c (http://www.dsmz.de) to cultivate *M. concilii*, at 37°C in Hungate tubes (Dutscher, Issy-les-Moulineaux, France) under a 2-bar H_2_/CO_2_ (80/20) atmosphere under stirring. *M. luminyensis* CSUR P135T was cultivated using *Methanobrevibacter* medium (medium 119: http://www.dsmz.de) modified by the addition of methanol and selenite/tungstate solution under 2-bar of H_2_/CO_2_ (80/20) atmosphere under stirring [4].

### Biocides and Squalamine Derivatives

Chlorhexidine (MP Biomedicals, Illkirch, France) and peracetic acid (ANIOS, Lille-Hellemmes, Fance) were tested in this study. Concerning the synthesis of squalamine derivatives, all the solvents were purified according to reported procedures and the reagents used were commercially available. Methanol, ethyl acetate, dichloromethane, ammonia and petroleum ether (35–60°C) were purchased from Solvants Documentation Synthèses (Peypin, France) and used without further purification. Column chromatography was performed on silica gel (70–230 mesh). ^1^H NMR and ^13^C NMR spectra were recorded in CDCl_3_ on a Bruker AC 300 spectrometer working at 300 MHz and 75 MHz, respectively (the usual abbreviations are used: s: singlet, d: doublet, t: triplet, q: quadruplet, m: multiplet). Tetramethylsilane was used as internal standard. All chemical shifts are given in ppm. A mixture of the desired ketosterol 1 or 2 (0.78 mmol), titanium (IV) isopropoxide (302 µL, 1.03 mmol) and the desired amine (2.34 mmol) in absolute methanol (5 mL) was stirred under argon at room temperature for 12 hours. Sodium borohydride (29 mg, 0.78 mmol) was then added at −78°C and the resulting mixture was stirred for an additional 2 hours. The reaction was then quenched by adding water (1 mL) and stirring was maintained at room temperature for 20 minutes. The resulting inorganic precipitate was filtered off over a pad of Celite and washed with Et_2_O and ethylacetate. The combined organic extracts were dried over Na_2_SO_4_, filtered and concentrated in vacuo. The crude product was placed in 10 mL of a MeOH/CHCl_3_ (1/1) solution, 128 mg of K_2_CO_3_ (0.93 mmol) were added and the mixture was placed under stirring for 24 h. The solvents were evaporated and the mixture was extracted with water and ethylacetate. The combined organic extracts were dried over Na_2_SO_4_, filtered and concentrated *in vacuo.* Subsequent purification by flash chromatography on silicagel (eluent: CH_2_Cl_2_/MeOH/NH_4_OH(32%), 7∶3∶1) led to a pale yellow solid. All squalamine derivatives reported here were prepared by using procedures similar to those described above and all the NMR and MS analyses were in accordance with the expected data.

### Testing Archaeacidal Activity

**Figure 1:**
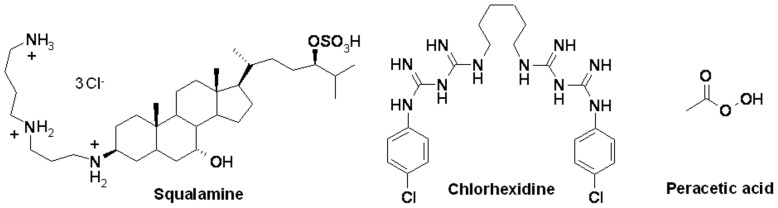
Structure of squalamine, chlorhexidine and peracetic acid.

**Figure 2:**
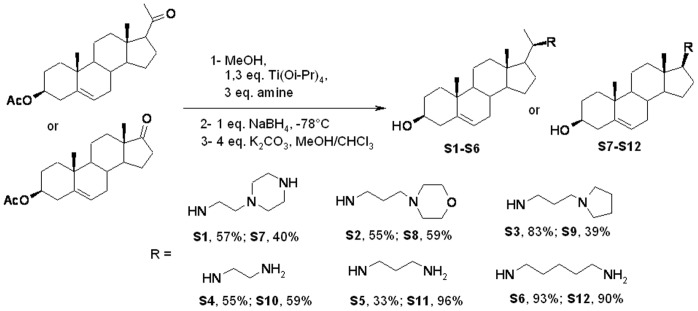
Overview of the pathway for the synthesis of squalamine derivatives S1–S12.

A filtered aqueous solution of each one of the 12 biocides (Figures 1, 2) was anaerobically added at a final 5 mg/L concentration into Hungate tubes [19] containing distilled water; tubes were previously sterilized by autoclaving at 120°C for 30 min under an H_2_/CO_2_ (80/20) atmosphere. The *in-vitro* archaeacidal activity of the biocides was determined by transferring 10E+05 archaea cells/mL of an exponentially growing culture into 4.5 mL of fresh medium containing 0.01, 0.05, 0.1, 0.2, 0.4, 0.8, 1, 5 or 10 mg/L of biocide. Tubes were incubated at 37°C under stirring and archaea growth was observed after a 5-day incubation. Cultures were centrifuged at 11,000 g for three minutes at room temperature, washed with fresh medium to remove traces of biocide and reinoculated into a new culture medium. Control cultures without biocide were incubated in parallel. Growth of archaea was assessed by optical microscopy observation and parallel measurement of methane production using a GC-8A gas chromatograph (Shimadzu, Champs-sur-Marne, France) equipped with a thermal conductivity detector and a Chromosorb WAW 80/100 mesh SP100 column (Alltech, Carquefou, France). N_2_ at a pressure of 100 kPa was used as the carrier gas. The detector and the injector temperature was 200°C and the column temperature was 150°C. The *in-vitro* activity of biocide under these culture conditions was verified as follows. Culture media 119, 322, 334 (http://www.dsmz.de) and *M.* *luminyensis* medium were supplemented with a final concentration of 0.01, 0.05, 0.1, 0.2, 0.4, 0.8, 1, 5 or 10 mg/L of biocide and were incubated at 37°C in a H_2_/CO_2_ (80/20) atmosphere for 10 days. The activity of each biocide was controlled using clinical isolates of *Escherichia coli* and *Staphylococcus aureus* [17], [20] in the same culture conditions as the tested archaea. Growth controls with appropriate media instead of derivative dilutions were introduced in all experiments. The minimal archaeacidal concentration (MAC) was defined as the lowest biocide concentration killing archaea organisms. This was measured by observing the inhibition of methane production and the absence of microscopically visible growth of this archaea.

## Results

The activity of the tested biocides incubated at 37°C under an H_2_/CO_2_ (80/20) atmosphere was confirmed by observing the killing of *E. coli* and *S. aureus* strains used as controls, after a 5-day incubation. All the positive control cultures of *M. smithii*, *M. oralis*, *M. arboriphilicus, M. stadtmanae, M. luminyensis*, *M. beijingense* and *M.* *concilii* incubated without biocide grew as expected with a methane production starting at day 3. As for four *M. smithii* strains, *M. oralis*, *M. arboriphilicus, M. concilii* and *M. beijingensis,* MAC was of 0.05 mg/L for squalamine derivative S1; 0.5 mg/L for squalamine and derivatives S2–S6; and 2 to 5 mg/L for derivatives S7–S12. As for *M. stadtmanae* and *M. luminyensis,* MAC was of 0.1 mg/L for S1; 1 mg/L for squalamine and derivatives S2–S6; and 5 mg/L to ≥10 mg/L for derivatives S7–S12. Chlorhexidine and peracetic acid lead to a MAC of 0.2 and 1.5 mg/L, respectively against all tested archaea (Table 1).

**Table 1:** Minimal archaeacidal concentration (MAC, µg/mL) of 15 biocides against archaea strains.

	S1	S2	S3	S4	S5	S6	S7	S8	S9	S10	S11	S12	Squalamine	Chlorhexidine	Peracetic acid
*M. smithii* ATCC35061^T^	0.05	0.5	0.5	0.5	0.5	0.5	5	5	5	5	5	2	0.5	0.2	1.5
*M. smithii*DSMZ 2374	0.05	0.5	0.5	0.5	0.5	0.5	5	5	5	5	5	2	0.5	0.2	1.5
*M. smithii*DSMZ 2375	0.05	0.5	0.5	0.5	0.5	0.5	5	5	5	5	5	2	0.5	0.2	1.5
*M. smithii*DSMZ 11975	0.05	0.5	0.5	0.5	0.5	0.5	5	5	5	5	5	2	0.5	0.2	1.5
*M. oralis*DSMZ 7256	0.05	0.5	0.5	0.5	0.5	0.5	5	5	5	5	5	2	0.5	0.2	1.5
*M. arboriphilicus*	0.05	0.5	0.5	0.5	0.5	0.5	5	5	5	5	5	2	0.5	0.2	1.5
*M. beijingense*DSMZ 15999	0.05	0.5	0.5	0.5	0.5	0.5	5	5	5	5	5	2	0.5	0.2	1.5
*M. consilii*DSMZ 2139	0.05	0.5	0.5	0.5	0.5	0.5	5	5	5	5	5	2	0.5	0.2	1.5
*M. stadtmanae*ATCC 43021^T^	0,1	1	1	1	1	1	10	10	10	10	10	5	1	0.2	1.5
*M. luminyensis*CSUR P13^T^	0.1	1	1	1	1	1	10	10	10	10	10	5	1	0.2	1.5

## Discussion

Although several reports have documented the presence of archaea in the human gut microbiota, they remain a neglected field in medical microbiology. This is illustrated by the complete lack of study addressing the archaeacidal activity of biocides. This is surprising considering intestinal archaea can potentially contaminate medical devices such as colonoscopes. Moreover, it has been shown that a purge prior to colonoscopy does not eliminate archaea from the gut [13]. Indeed, non-methanogenic halophilic archaea have been detected in one purge preparation [13]. Accordingly, transmission of methanogenic archaea between patients via reusable medical equipments such as colonoscopes may alter the intestinal microbiota, causing pathologies such as digestive tract diseases and obesity [21], [22]. Studying the archaeacidal activity of some biocides is even more urgent, as archaea exhibit a unique cell wall structure and composition which keep the results from being simply extrapolated from what was already known for bacteria. Accordingly, in general, archaea are more resistant to antibiotics than bacteria [14], [15].

Therefore, there was a need to assess the archaeacidal activity of biocides used in routine. Here, since the controls introduced in all experiments produced the expected results, the reported data have been interpreted as authentic. In particular, we controlled the *in-vitro* activity of molecules herein tested under the unusual atmosphere comprising of 80% H_2_ and 20% CO_2_ required for growing methanogenic archaea. In addition, we tested squalamine as a positive control molecule and observed a MAC value in the range of the previously reported value [17], [20]. In the present study, we extended data on squalamine to *M. arboriphilicus, M. beijingense* and *M. concilii* which have not been previously tested for their susceptibility to squalamine.

We observed that the susceptibility of archaea to biocides varies depending on both the archaea species and the nature of the tested biocide. In particular, *M. stadmanae*, an archaea found in almost one-third of individuals [10], was twice more resistant to biocides than the other tested archaea. This observation is in line with our previous observation that *M. stadmanae* is more resistant to antibiotics than *M. smithii* [14], [15]. The mechanisms underlying such differences are unknown but they should be broad-spectrum, relatively poorly specific mechanisms such as differences in the cell wall composition. Archaea possess membranes made of chemically stable glycerol-ether lipid bonds. In some archaea the lipid bilayer is replaced by a monolayer, in which the tails of two independent phospholipid molecules are fused into a single molecule with two polar heads [23]. This fusion may render membranes more rigid and stable in harsh environments [24]. Archaea lipids are based upon long isoprene side chain and often cyclopropane or cyclohexane rings. These branched chains may keep archaeal membranes from leaking at high temperatures or help them resist to disrupting membrane agents [25], [26]. Another broad-spectrum mechanism relies on efflux of molecules. Whereas the *M. smithii* ATCC 35061 complete genome (GenBank accession number CP000678) encodes for six efflux pumps representing 3/1000 of the genome size, *M. stadtmanae* DSM 3091 complete genome sequence (GenBank accession number NC 007681) encodes for four efflux pumps representing 4/1000 of the genome size.

**Figure 3:**
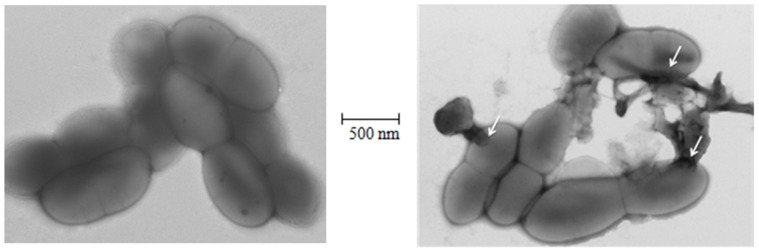
Images by electron microscopy demonstrating the morphological effects of squalamine on *M. smithii* cell wall. (A) *M. smithii* without squalamine. (B) *M. smithii* +1 mg/L squalamine. White arrows show holes caused by squalamine on *M. smithii* cell wall. The scale bar corresponds to 500 nm.

Two commonly used biocides chlorhexidine and peracetic acid, exhibited an archaeacidal activity under current decontamination protocols and by using concentrations of 0.2 and 1.5 g/L, respectively. However, squalamine and its derivatives exhibited a higher activity than these two usual biocides on the majority of here tested archaea, not on all archaea. Interestingly, these compounds are equally active against Gram-negative and Gram-positive bacteria, including bacteria from the human intestinal microbiota [17], [20]. They act directly on the cell membrane of the bacteria by creating holes (Figure 3), emptying the cell cytoplasms which lead to the death of the bacteria [17]. Data herein reported indicate that some squalamine derivatives exhibited an increased *in-vitro* activity against methanogenic archaea, particularly for derivative S1. Indeed, the structure of the different squalamine derivatives greatly influences their archaeacidal activity: aminosterol derivatives from dehydroepiandrosterone (DHEA) demonstrate a lower activity (around 5 µg/mL) compared to pregnenolone derivatives (0.05 to 0.5 µg/mL) while they differ only by the length of the side chain in position 17 suggesting a required specific conformation by targeting archaea. On the other hand, even in the same series the activity is conserved whatever the nature of the amino side chain introduced except for derivative S1 which is ten times more active and which differs only by the presence of three positive charges instead of two in all the other products suggesting a potent interaction of the positive charge of the compound with the negative charge of the archaeal membrane (Figure 1). All these features constitute a basis for the development of a new class of biocides devoted to the decontamination of archaea-contaminated medical devices.

### Conclusion

The data reported here indicate that peracetic acid, which is routinely used for the desinfection of medical devices including colonoscopes, is effective against human-associated archaea. Nevertheless, less corrosive agents such as squalamine and its parent derivatives appear as better promising biocides against the majority of human-associated archaea. Studies are now under current investigation to understand their involved mechanistic rationale and improve their potent routinely use for disinfection of medical devices including colonoscopes.
