# Correction: *In-Vitro* Archaeacidal Activity of Biocides against Human-Associated Archaea

**DOI:** 10.1371/annotation/917c9687-a3c4-4e82-a5d4-4424f8f1de34

**Published:** 2013-07-02

**Authors:** Saber Khelaifia, Jean Brunel Michel, Michel Drancourt

The name of the second author was given incorrectly. The correct name is: Jean Michel Brunel. 

The correct citation is: Khelaifia S, Brunel JM, Drancourt M (2013) In-Vitro Archaeacidal Activity of Biocides against Human-Associated Archaea. PLoS ONE 8(5): e62738. doi:10.1371/journal.pone.0062738. The name is correctly reflected in the abbreviation in Contributions as it is. 

